# Hemorrhagic Malarial Fever

**Published:** 1874-09

**Authors:** Wm. J. Johnson

**Affiliations:** Fort Gaines, Ga.


### HEMORRHAGIC MALARIAL FEVER.

By WM. J. JOHNSON, M.D., Fort Gaines, Ga.

So far as my observation has extended, this is a disease upon which but little has been written by our medical authors. When we reflect that the disease is justly regarded as one so dangerous and fatal to human life, we are amazed at the fact, and confess we are at a loss to account for it. Actuated by a spirit similar to that which moved the poor widow of whom we read in Holy writ, who cast her "mite" into the treasury of her Lord, I voluntarily bring forward this my humble offering, and cast it as my "mite" into the repertory of practical medicine.

Hemorrhagic malarial fever, or malarial hematuria, is generally recognized by the non-professional by the various synonyms of "yellow disease," "bloody jaundice," "yellow fever," etc.; although it differs from the former in almost every essential and pathognomonic characteristic, except the intense yellow color of the entire cutaneous surface. The disease derives these appellations from the circumstance of its being uniformly characterized by an intensely icterode appearance of the skin in every part of the body, being of a deep yellow or saffron hue, and accompanied by profuse discharges of bloody urine (hæmaturia.)

Its frequent appearance within the past few years in the malarious portions of the Southern States, especially in Florida, Alabama, Mississippi, Louisiana, and some portions of our own State, together with the frightful ratio of mortality which accompanies its invasions, has very properly awakened in the minds of Southern physicians an anxious and commendable spirit of inquiry into its causes, pathology and treatment. In regard to the causes which produce this disease, and its pathological phenomena, there is among the Southern profession no disagreement of opinion as regards the most successful plan of treating it.

Unlike yellow fever, which occurs only in warm climates and hot weather, and to which a speedy stop is put by a killing white frost, this disease is as often seen in mid-winter as at any other season of the year. A very large proportion of the cases which I have been called upon to treat have occurred in the months of November and January. Unlike yellow fever, it never prevails epidemically, neither is it in the least degree contagious or infectious, though superstitious and ignorant people have a great horror of it, and feel strongly disinclined to approach a patient with the disease, for fear of contracting it. Unlike yellow fever, its invasions are not confined to maritime cities and large towns, nor to the vicinity of large streams and their tributaries entering into the Gulf of Mexico, or the Atlantic Ocean, but it occurs sporadically and at any season, in subjects who are predisposed to its attacks by having had their constitutions impaired, undermined or broken down by previous and frequently recurring attacks of intermittent fever. The only persons who are liable to be attacked by hemorrhagic malarial fever are those who for weeks or months have suffered with frequent relapses of intermittent fever, and neglected to arrest them until several consecutive paroxysms have taken place, followed by relapses every few days, until the chills assume a chronic type, manifested by symptoms of general cachexia, indicated by derangements of the chylopoietic viscera, such as sallow complexion, enlarged, torpid, or otherwise diseased liver; enlarged, indurated, or inflamed spleen; capricious or morbid appetite; lassitude, want of energy, and a general cadaverous appearance of the patient.

There are four prominent symptoms which particularly characterize this fever, and which are always invariably present, to-wit: 1st. The chill or ague, which precedes the fever. 2d. Excessive nausea and vomiting. 3d. Intense icterode appearance of the entire cutaneous surface. 4th. Copious evacuations of bloody urine. Besides these four prominent symptoms there are a few other which I will now notice. Among these are pain in the epigastric region, and extreme thirst. Towards the last stage, in fatal cases, the patient has an anxious expression of countenance, indicative of fear and extreme nervous suffering. By degrees he becomes gloomy, despondent and taciturn, and apparently indifferent to his fate, as though he was bordering on "horrid despair;" and towards the last stage the matters ejected from his stomach bear a striking similarity, if not an exact resemblance, to coffee grounds, or the black vomit of yellow fever. On the 2d day of last December I lost a patient who only lived about twenty-four hours after he was attacked with this disease, and who but a few minutes previous to his dissolution ejected from his stomach a substance strongly resembling coffee grounds. The chill or ague is suddenly ushered in with severe shaking rigors, especially in violent attacks. These rigors are marked by an intensity of degree far beyond those which occur in ordinary chills, and in many cases are prolonged much beyond the length of time generally occupied by the cold stage of an intermittent fever. The chill is attended with an inordinate, yea, insatiable, thirst; the patient constantly calling for water; the stomach constantly rejecting that fluid immediately, even though it be swallowed in the most minute quantities. The cutaneous surface now begins to assume an intensely yellowish appearance, which pervades in a short time the entire body. The desire to micturate becomes urgent, and the patient, at the first and subsequent attempts at micturition, voids large quantities of bloody urine. The hemorrhage from the bladder persists in a frightful manner, until the disease, in fatal cases, terminates in death; and in more favorable cases until restrained by appropriate remedies. The nausea, vomiting and gastric irritability, which are the most intractable symptoms with which the physician has to contend, are not only annoying to the patient, but distressing to witness, accompanied, as they always are, by the most tormenting thirst.

In many instances the reaction from the chill is tardy, imperfect and incomplete; in others there is no reaction whatever. The case of John Reynolds, of Dougherty county, who died a few weeks ago, is a case which conclusively verifies this assertion. The *Albany News*, in the announcement of his death, stated that Reynolds was suddenly seized with a chill; in fifteen minutes hemorrhagic malarial fever developed itself, and in five hours thereafter he was a corpse. This case furnishes conclusive evidence of the overwhelming power of the disease. Here the force of the blow was so violent, and the vital energies of the victim so feeble, that the constitution of the patient could offer no effectual resistance, and was forced, in the short space of five hours, to succumb to his fate, and give up the ghost. In this, as well as many other cases which I have witnessed, no reaction could possibly have taken place.

Pathological deductions may be drawn from the following facts in regard to the true nature of the disease: The frequent repetition or relapses of intermittent fever are associated with engorgement or congestion of many of the viscera of the three great cavities of the body, and are followed by pathological changes in these organs of a complex and complicated nature. In some cases, owing to the mildness of the attack, these changes are nothing more than a mere disturbance of the functions of those organs, but in others, especially in protracted cases, they become structural, or organic. An intelligent physician, conversant with the pathological phenomena of a chill, or the cold stage of an intermittent fever, will have observed that in this stage the centripetal predominates over the centrifugal vital force; the blood in the cold, or chill stage, receding from the pheriphery of the body to the internal viscera, producing a state of temporary congestion therein, and, for the time being, throwing the circulation off its balance, and thus suspending its equilibrium. When reaction ensues, producing the hot stage, the blood and other vital fluids which, during the cold stage, had been driven by the centripetal force to the internal organs, leaving the surface cool, the skin shrivelled, and the internal organs, for the time being, in a state of engorgement or congestion, are now, by means of the centrifugal force, restored again to a state of equilibrium; the reaction following these phenomena culminating in fever, more or less intense, according to the violence of the reaction. This is the usual course of these two stages of an intermittent fever; but in some malignant cases there is scarcely any reaction whatever; the patient, if old and intemperate, with broken down constitution from previous disease and habits, dying in the cold stage from the violence of the attack and the want of ability in the vital forces to resist the cause. In the case of Reynolds, above mentioned, whose constitution was no doubt seriously impaired by frequent relapses of chronic chills, there could have been no reaction; death ensuing in so short a space of time from the violence of the attack and the feeble powers of resistance.

While the reaction in some cases is tardy and imperfect, in others again it is too violent; being not unfrequently attended by local determinations of blood or by inflammation of the affected organs. These local inflammations being aggravated and intensified with each recurring paroxysm of chill, ultimately produce structural changes or indurations; the consequence is they react on the whole system, and instead of being, as at the commencement, merely the effects, now are the cause of the continuance of the disease; at least they have a tendency to protract, aggravate and intensify the original malady, until, after some time, owing to the state of the viscera, disease of an intricate and complicated character is developed, such as the one now under consideration, resulting in a disordered condition or modification of the secretory or nutritive processes.

To sum up in a few words its pathological characteristics, the disease termed hemorrhagic malarial fever, or malarial hematuria, is nothing more, nothing less, than the grave pathological changes induced by the repeated and constantly recurring paroxysms or relapses of chronic intermittent fever, acting and reacting on the vital organs, or those essential to the existence of life, until some important lesion supervenes, either in the liver, spleen, kidneys, stomach, mucous coat of the intestines, or the cerebro-spinal system of nerves. Expressed briefly, in as few words as possible, this, in my judgment, is the true pathology of hemorrhagic malarial fever.

I will now take a brief pathological review of some of the prominent symptoms of the disease; and first of the nausea and vomiting. The intensity of these symptoms, which invariably accompany the disease, is due to the vehement engorgement or congestion of the stomach, particularly its mucous coat. The great accumulation of the circulating fluids in the internal organs, like a heated furnace, generates an immense amount of internal heat, and this creates an urgent desire for cold water; hence the inappeasable thirst of which the patient incessantly complains. The vomiting is not merely sympathetic, as in certain affections of the brain, kidneys or uterus, induced purely by nervous influences, but it is symptomatic of engorgement, or extreme hyperæmia of the stomach, and in some cases of gastric inflammation. The vomiting and thirst are the most intractable and rebellious symptoms to control, and are absolutely unmanageable by the usual anti-emetic remedies employed to arrest vomiting. Every thing taken into it offends the stomach, and causes it to reject indiscriminately all kinds of fluids in a short time after they have been swallowed. The hematuria is not only symptomatic of the pathological changes already described, but, in addition thereto, there is congestion of the kidneys and bladder. The hemorrhage from the bladder is active, the patient in every act of micturition voiding large quantities of bloody urine. He does not complain of much pain in the region of the bladder until the exhaled fluid distends the viscus, and the desire to urinate becomes distressingly urgent, though a dull aching pain in the lumbar region is almost constantly experienced. When the bladder becomes distended, the pain in that organ is excruciatingly painful, and the patient calls impatiently for the urinal, as though unable to tolerate or endure the presence of the urine in the viscus for a moment longer. The act of micturition is generally attended with straining efforts, and the evacuation of the bladder is sometimes followed by a sense of faintness.

The icterode appearance of the cutaneous surface makes its appearance in the early stage of the disease, but deepens with its progress, until it assumes a deep lemon or saffron-colored hue. In this respect it differs from yellow fever; the jaundiced appearance of the skin in that disease not appearing before the third or fourth day, until the fever has reached the second stage. The icterode appearance of the skin is a prominent or pathognomomic symptom of the disease, being invariably present, and is, perhaps, attributable to duodinitis, as the patient frequently complains of pain in the epigastric or duodinal region; augmenting, through sympathy, the secretion of the bile, and preventing its discharge through the ductus-communis-choledochus; or, like jaundice, it may be produced by any cause which could, directly or indirectly, interfere with or obstruct the flow of bile in its passage from the liver into the intestine, and thus cause it to be thrown into or diffused through the circulation.

In regard to the diagnosis of malarial hematuria, or hemorrhagic malarial fever, the peculiar and distinct features of the disease are so well marked and unmistakably stamped upon the patient as to render a mistake in this respect almost impossible.

The indications pointing to a favorable termination of the disease are marked by an arrest of the nausea and vomiting, subsidence of the fever, disappearance of blood in the urine, the yellow appearance of the skin beginning to vanish, etc.

So far as my experience and observation have extended, the negro population enjoy an immunity, amounting almost to an exemption, from this disease. I cannot now call to mind a single case which I have ever treated among that race of people. In this respect there is a striking similarity to yellow fever. Dr. Cartright, late of New Orleans, a gentleman of profound research, extended observation and enlarged experience, whose opinion on this subject is entitled to much weight with the profession, and who was enabled to speak almost *ex cathedra,* declared that the negro population is almost, if not entirely, exempt from the invasions of epidemic yellow fever. He saw and treated many cases of that disease, both in New Orleans and Natchez, amongst the white, but saw no case among the colored population.

This opinion of Cartright has been confirmed by the experience of physicians practicing in the West India Islands, Mobile, Charleston, Portsmouth, Norfolk, and other seaport cities having a large proportion of colored population, and where the disease has appeared in its most malignant and virulent epidemic forms. It is possible that since the negro has, by Congressional enactment, been invested with the rights of citizenship he may be liable to contract this disease. This suggestion is predicated upon information which I received from a gentleman a few days since—a gentleman truthful and well informed upon the subject. He assured me that during the prevalence of the fearful epidemic—*i. e.*, yellow fever—which scourged the inhabitants of the city of Shreveport last fall, being then himself an eye witness, he saw and knew of a number of cases of that disease amongst the negroes, and that it was quite as fatal amongst that as the white race. Whilst the political and social status of the negro have been changed by statutory enactment, is it not possible for his natural, physical and pathological status to undergo some mysterious and unaccountable changes also?

The prognosis of the hemorrhagic malarial fever, if the patient is seen in time by the medical attendant, and skilfully treated by appropriate remedies, is not so grave as many persons suppose, but if not skilfully treated it is one of the most dangerous complaints endemic in this climate. Yet, if the proper remedies be applied in time, it readily yields to medication, and becomes very amenable to treatment. In the treatment of this disease there are four indispensable therapeutic agents, which I generally employ, and which, from the success that attends my practice, I am satisfied to retain as my *armamentarium medicum* in its management. These are, first, opiates; second, spirits turpentine; third, quinine; fourth, potassic iodide. These, with the addition of some minor adjuncta, constitute every thing needful for successfully treating hemorhagic malarial fever.

I will now speak of the therapeutic value of each of these agents, in the order in which they have been named, and first of opiates. The most intractable and rebellious symptoms with which the physician has to contend, in the treatment of this disease, are nausea, vomiting and gastric irritability. Without the use of opium and its preparations all the efforts of the physician to control them would prove abortive and unavailing. The anti-emetic remedies in common use, and the anile preparations generally resorted to would be wholly inert, but opium is the remedy to meet this indication *par excellence.* When judiciously prescribed and administered it never fails to control these troublesome symptoms. It should be hypodermically used in the form of a solution of morphine of the strength of one-third of a grain to each injection, and repeated, if necessary, until the gastric irritability be allayed, even if it be continued to the extent of blunting the sensibilities of the stomach by producing partial narcotism. In the absence of a hypodermic instrument large doses of laudanum, in the form of enemata, mixed with a little prepared starch, or thin mucilage, will answer as a substitute, or laudanum administered by the mouth, repeated as often as rejected, until this organ will tolerate its presence and retain it. As a valuable adjutor to the opiates, sinapisms should be applied extensively over the epigastric region, as well as to the spinal column and extremities. Cold water, taken even in the smallest quantities, proves a violent irritant to the stomach, which will be rejected soon after it is swallowed, preceded by strong and straining efforts, retching, etc. Therefore we must enjoin the most absolute abstinence from the use of this and other fluid substances until we get the troublesome and distressing symptom under control. The first thing, then, in the treatment is to control the nausea and vomiting; otherwise no medicine administered by the mouth will be of any avail to the patient, as the stomach will reject it as soon and as often as swallowed until this *desideratum* is accomplished. Having, by the means indicated, succeeded in arresting the vomiting, and quieted the stomach sufficiently to retain medicine, we are then prepared to attack the disease with the emulsion of spirits turpentine, quinine and potassic iodide, and such other adjuvants as we may deem applicable to the treatment of the disease.

The next thing to be done in the treatment is to arrest the hematuria. To attain this end we immediately prepare an emulsion of spirits turpentine, which we usually make by beating up the yolk of an egg with sugar, adding a little carbonate of soda, and then the spirits turpentine with a small quantity of comp. spts. lavender, and triturating the whole together until the ingredients are thoroughly incorporated. The emulsion should be sufficiently strong to contain twelve or fifteen drops of the turpentine in each dose, and should be repeated every three hours. From the well known effects of ergot in restraining hemorrhage, by its action on the vaso motor nerves and on the spinal cord, especially in arresting hemorrhage from the lungs, uterus, bladder and bowels, the fluid extract of that article, or a strong tincture, might be a valuable adjunct to the turpentine, but I have never found it necessary in my practice to substitute any other medicine in lieu of the turpentine, or to add anything thereto, with a view to increase its efficiency. I regard its hemostatic properties in this disease so potent as to amount almost to a specific. I know of nothing better in the treatment of all internal hemorhages from its constringing properties on the capillary vessels of the mucous membranes. Some of our physicians use tannin, acetate of lead, alum and other astringent substances in place of the turpentine, but there is nothing belonging to this class of remedies upon which so certain and uniform reliance can be placed to accomplish the desired object as this article.

The next great remedy in the treatment of this disease is the sulphate of quinine. This agent performs an indispensable part in its treatment. It should be exhibited in large doses; say from six to eight grains every two hours, until the pulse and temperature are brought down to a normal standard. It must be commenced with, fever or no fever, as soon as the stomach will retain it; waiting for no intermission or remission of the febrile symptoms. Give it during the pyrexial as well as the apyrexial stage of the disease. If the stomach cannot tolerate its presence, it must be administered in the form of enema, in the dose of fifteen or twenty grains, suspended in a little mucilage, with a few drops of laudanum. Violence of the fever during the paroxysm and congestive engorgement of the liver or spleen, are not, as some suppose, a contraindication to its administration. The most desirable form of exhibiting the quinine is the pilular, except in cases of children and those persons whose peculiar idiosyncrasy of constitution prevents them from swallowing pills. Quinine increases and exalts the tone of the muscular fibres and of the fibre of the vessels; hence, by its use the pulse becomes fuller, stronger and more regular, and the muscular power is increased, which leads to an augmentation of the vital energies of the whole system.

An intelligent and well-informed lady, residing in Leon county, Florida, assures me that the physicians in that section of the State have discarded the use of quinine entirely in the treatment of this disease, and substituted in lieu of it the ferrum sesqui-cyanidum, (or blue iron.) On account of its insipidity and smallness of dose, and the fact that it does not disagree with the most irritable stomach, it is peculiarly adapted for children. It has long been a favorite remedy with some of the German physicians in the treatment of intermittent fever, who believe it to be as certain, prompt and efficacious in its effects as the preparations of cinchona. Will not some of the Florida physicians be induced to publish, in some of our medical periodicals, their detailed experience with this article?

We will now speak of the potassic iodide. I generally administer it conjointly with quinine, as soon as the irritability of the stomach is sufficiently controlled to enable the patient to retain it. I dissolve sixty grains of the iodide in twelve tablespoonfuls of water, and give a tablespoonful at a dose, every three hours, persistently, day and night, until symptoms of amelioration in the disease occur. Then lengthen the interval to every six or eight hours; at the same time administering the quinine as before mentioned, until the pulse and animal temperature are brought down to a normal standard. In some cases enormous quantities of quinine, as from sixty to one hundred grains or more, will be required before this object can be accomplished. As soon, however, as this end has been attained, the quinine must be suspended and the iodide continued until the disease shall have yielded.

This agent is a powerful deobstruent and alterative, and by its liquifacient and dissolvent powers, possesses far more value than some physicians, who are ignorant of its wonderful effects, will concede to it. It is, and for a long time has been, a favorite remedy with me in the treatment of almost all visceral complaints of a glandular character, such as chronic hepatitis, chronic splenitis, and all those organic and functional disturbances of the abdominal viscera which are perceptible in a state of general cachexia, especially when it is combined with some of the preparations of iron; the citrate, potassio tartrate, or tincture of the chloride, I prefer over all others. In obstinate chronic intermittents, where the relapse is certain to occur periodically every seventh or fourteenth day, and where all the prophylactic remedies usually resorted to, such as quinine, arsenic, *et id omne genus* fail, this agent will be found a most valuable and effective remedy.

I had intended, when I began to write this article, to have illustrated the treatment of hemorrhagic malarial fever by reporting in detail a number of cases which I have successfully treated by the plan described in the foregoing article, but as this paper has been extended to a much greater length than I originally designed, it might perhaps be irksome to the reader to follow me through the prolixity of detail, which would necessarily accompany a minute or particular narration of the cases.

